# CT Fluoroscopy-Guided Percutaneous Gastrostomy in the Palliative Management of Advanced and Relapsed Ovarian Cancer: The Charité Experiences and a Review of the Literature

**DOI:** 10.3390/cancers15184540

**Published:** 2023-09-13

**Authors:** Emel Canaz, Jalid Sehouli, Bernhard Gebauer, Laura Segger, Federico Collettini, Timo Alexander Auer

**Affiliations:** 1 Charité Comprehensive Cancer Center (CCCC), European Competence Center for Ovarian Cancer (EKZE), Department of Gynaecology and Gynaecological Oncology, Charité-University Medicine of Berlin, Campus Charité Virchow, 13353 Berlin, Germany; jalid.sehouli@charite.de; 2 Department of Radiology, Charité-University Medicine of Berlin, Campus Charité Virchow, 13353 Berlin, Germany; bernhard.gebauer@charite.de (B.G.); laura.segger@charite.de (L.S.); federico.collettini@charite.de (F.C.); timo-alexander.auer@charite.de (T.A.A.); 3 Berlin Institute of Health (BIH), Anna-Louisa-Karsch 2, 10178 Berlin, Germany

**Keywords:** ovarian cancer, malignant bowel obstruction, palliative care, CT-guided percutaneous gastrostomy

## Abstract

**Simple Summary:**

Malignant bowel obstruction (MBO) requires adequate palliation in progressive ovarian cancer. Percutaneous endoscopic gastrostomy has limitations in 32.7% of patients with advanced/recurrent ovarian cancer due to anatomical constraints and a lack of transillumination. CT-guided gastrostomy is a safe and effective procedure, enabling rapid recognition of the anatomy, particularly in complex patients with peritoneal carcinomatosis and previous multivisceral surgeries. This technique should be particularly emphasized within the palliative management of MBO. A clinical registry should be implemented to evaluate the effectiveness of various treatment strategies in MBOs associated with gynaecological cancers.

**Abstract:**

Peritoneal carcinomatosis-associated malignant bowel obstruction is a common feature that merits more attention in advanced and recurrent ovarian cancer. Decompressive gastrostomy is one of the most preferred methods to palliate distressing symptoms and maintain patients’ quality of life. We retrospectively identified 31 patients with ovarian cancer-associated MBO, who underwent decompressive CT fluoroscopy-guided percutaneous gastrostomy (CT-PG) between September 2015 and April 2023 at our institution. A systematic literature review was conducted for CT-guided gastrostomy in ovarian cancer. Prior to CT-PG, 27 (87%) patients underwent unsuccessful attempts at endoscopic gastrostomy or surgery due to bowel obstruction; a total of 55% had received ≥3 lines of chemotherapy. CT-PG could be successfully inserted in 25 of 31 (81%) patients without grade 4–5 complications. CT-PG insertion was feasible in 76% of patients with previous unsuccessful attempts of endoscopic gastrostomy. A total of 80% of patients with a successful insertion had considerable symptom relief and could tolerate fluid intake. Mean survival after the procedure was 44.4 days. Chemotherapy could be administered in 7 of 25 (28%) patients following the CT-PG insertion. CT-guided percutaneous gastrostomy is a safe procedure that effectively manages intractable symptoms of bowel obstruction in ovarian cancer. This minimally invasive technique should be emphasised as a routine instrument within the palliative management of MBO.

## 1. Introduction

Efforts in ovarian cancer mainly focus on therapeutic options and the curative management of the disease with multivisceral surgeries, novel targeted therapies and various investigations, which have also contributed to the survival of these patients in the last few years [1]. Nevertheless, disease recurrence is inevitable in approximately three-quarters of patients; hence, ovarian cancer is still the leading cause of gynaecological cancer-related death in Europe and in the US, ranking second in mortality worldwide [2,3,4]. When the disease progresses, the timely integration of palliative care measures is crucial to relieve the debilitating symptoms and maintain the quality of life. 

One common feature of progressive disease in ovarian cancer is peritoneal carcinomatosis-associated bowel obstruction. Compression of the alimentary tract with either a single metastasis or disseminated disease can result in mechanical ileus, but also functional disturbance is frequently observed due to the infiltration of intestinal nerves regulating bowel motility [5]. Troublesome symptoms of nausea, vomiting, and pain towards the end of life can be distressing for patients and their families. Counselling these patients and managing malignant bowel obstruction (MBO) deserve more attention in gynaecological oncology since MBO occurs quite frequently, in almost 25–50% of patients with advanced ovarian cancer [6,7].

The holistic and personalised concept of palliative care in MBO should embrace a great variety of aspects, from maintaining parenteral nutrition to palliative interventions such as the insertion of gastric and colorectal stents or decompressive gastrostomy. Palliative surgeries with stoma formation and bypassing anastomosis can also be the choice of treatment according to the level of obstruction. Nevertheless, surgery due to MBO is generally associated with high morbidity and mortality; hence, it should be decided individually after a critical evaluation [8,9]. 

Percutaneous endoscopic gastrostomy (PEG) is one of the most preferred methods to achieve intestinal decompression. Endoscopic gastrostomy requires an adequate diaphanoscopy during gastroscopy. Ascites and diffuse peritoneal carcinosis were regarded as relative contraindications with concerns of loss of transillumination, breakage, and leakage due to the tense abdominal wall [10]. In peritoneal carcinomatosis and previously operated patients in particular, it may be difficult to demonstrate a safe window to puncture, as the peritoneal tumour mass may obscure the underlying anatomy. Alternatively, CT-guided percutaneous gastrostomy (CT-PG), which optionally utilises fluoroscopic guidance, has been primarily advised in cases with unfeasible endoscopic insertion [11]. CT-PG is commonly described in oropharynx and oesophagus cancers with difficulties in utilising the endoscope, given the anatomical restrictions [12,13]. Despite decades of experience, CT-PG is rarely addressed as a purely palliative intervention in gynaecological cancers.

This article aims to present our Charité experiences in terms of the success rate, complications, symptom relief, ability to receive further chemotherapy, and survival in ovarian cancer-associated small bowel obstruction managed with CT-PG. Our primary goals were to estimate the feasibility, safety, and effectiveness of the procedure with regard to symptom palliation. We believe that the role of this minimally invasive technique should be separately highlighted within the palliative management of these patients.

## 2. Materials and Methods

In this descriptive study, we retrospectively reviewed the data of patients with malignant small bowel obstruction associated with ovarian cancer undergoing CT-PG during their treatment in the Department of Gynaecology, Charité Comprehensive Cancer Center, University of Berlin. CT-PG was performed in the Interventional Radiology Section of the Department of Radiology. The Institutional Review Board approved the study and waived informed consent because of the anonymised use of data and retrospective nature of the study. 

Patient demographics, clinical characteristics, disease status, interventional attempts prior to CT-PG insertion, failure rate and need for subsequent interventions, symptom relief, ability to receive further chemotherapy, complications, and postprocedural survival from the date of CT-PG were retrieved from institutional medical records. The diagnosis of malignant small bowel obstruction was made on a clinical and radiological basis (sonography, abdominal contrast X-rays) and confirmed by computerised tomography in all patients.

Success was defined as the correct intragastric placement of the gastric tube (G-tube). Relief of symptoms was documented according to the alleviation of nausea, vomiting, and abdominal pain on the basis of patients’ reports during multidisciplinary clinical assessments by attending physicians, nurses, and a palliative team including palliative care physicians, physiotherapists, psycho-oncologists, and nutritionists.

Complications were graded using the Common Terminology Criteria for Adverse Events (CTCAE) Protocol for Procedural Complications, version 5.0 [14]. Grades 4–5 were regarded as severe complications, such as procedure-related death or admission to the intensive care unit, and complications requiring surgical intervention. Moderate complications (grade 3) were defined as medically significant but not immediately life-threatening complications, such as the misplacement and extragastric positioning of the G-tube. Mild complications (grades 1–2) were defined as those requiring local or non-invasive interventions. Furthermore, complications that occurred within the 30 days were defined as early, and subsequent complications were defined as late complications. A postprocedural need for tube exchange due to functional disturbances related with tube clogging were also documented without grading as an interventional complication. 

**Figure 1:**
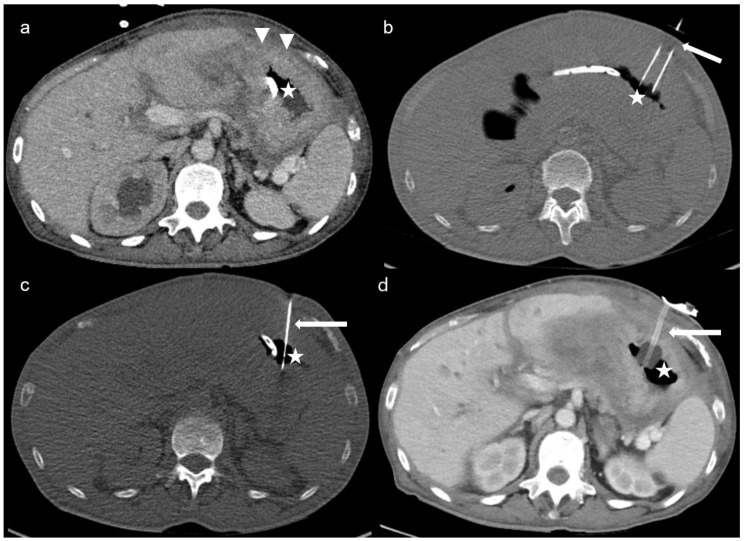
Multiple sequential axial-enhanced CT and unenhanced CT-fluoroscopy images: (**a**) triangles show thickened peritoneal surfaces due to carcinomatosis, which hinders diaphanoscopy at the endoscopic approach. The nasogastric tube is used to insufflate the stomach *(star)*; (**b**) the gastropexy device *(arrow)* passes through the abdominal wall and punctions the anterior gastric wall. Gastropexy sutures are applied 2 cm apart to fasten the stomach to the anterior abdominal wall; (**c**) an 18G access needle *(arrow)* is inserted in the air-filled stomach *(star)*, followed by the introduction of a 0.035″ Amplatz super stiff wire (Boston Scientific, Marlborough, MA, USA) and the insertion of a 16F peel-away sheath (Cook Medical); (**d**) a 15F gastric tube *(arrow)* is placed in the gastric cavity *(star)* with an inflated balloon (Freka Pexact CH/FR 15).

The technique of CT-PG was described in detail in a previous study of our institution, including all CT-guided gastrostomies with nutritional or decompressive purposes [15]. CT-PG was applied by the Seldinger technique under local anaesthesia with 1% lidocaine. In all patients, preprocedural sequential enhanced CT slices were acquired to demonstrate the anatomy, and CT-fluoroscopy was utilised for guidance. A gastropexy device was used to puncture the anterior gastric wall and to place the gastropexy sutures (Freka Pexact FR15; Fresenius Kabi, Bad Homburg, Germany). Figure 1 demonstrates the steps of the CT-PG in a case of peritoneal carcinomatosis, in whom endoscopic gastrostomy was not feasible due to inadequate transillumination.

Before and after the procedure, depending on complete or partial obstruction, pharmacological treatments including antiemetics, steroids, prokinetics or anticholinergics, and somatostatin analogues were used to assist with the symptoms at the discretion of the treating physician. Conservative management with nasogastric tube decompression and bowel rest was the initial step before referring gastrostomy in all patients. 

Descriptive statistics were used to analyse patient demographics and outcome measures. The results are expressed as the mean or median (range).

### Literature Review

A systematic review of the literature was conducted through PubMed for studies from 1991 to April 2023 pertaining to patients who underwent CT-guided gastrostomy for decompressive purposes in ovarian or peritoneal cancer. The following terms were used for the database search: ((CT-guided) OR (computed tomography)) AND ((gastrostomy) OR (gastric tube)) AND ((malignant bowel obstruction) OR (ovarian cancer) OR (peritoneal cancer)). Only English-written articles were selected for review.

The review of the literature using the above-mentioned search strategy in PubMed revealed 35 publications. Seventeen additional studies on percutaneous gastrostomy were included by examining the bibliographies of the studies to increase the coverage of the review. In the first step, titles with abstracts and then the main texts of 52 publications were reviewed with regard to the purpose of CT-guided gastrostomy and underlying disease. Expert opinions and reviews, studies that did not explicitly refer to the CT-guided insertion, radiological studies not providing a concrete number of patients regarding CT-guided gastrostomy, studies not including patients suffering from ovarian cancer, papers without full-text articles, and gastrostomies only for nutritional purposes were excluded. Six studies indicating CT-guided gastrostomy and including patients with ovarian cancer were selected for data extraction [13,16,17,18,19,20]. 

## 3. Results 

From September 2015 to April 2023, 143 patients with peritoneal carcinomatosis associated with ovarian cancer underwent decompressive gastrostomies via endoscopic (*n* = 107), surgical with/without combined endoscopic visualisation (*n* = 5), or CT-guided (*n* = 31) approaches. Secondary procedures for the reinsertion or correction of the gastric tube in patients who already had a gastrostomy and patients with other gynaecological or non-gynaecological malignancies were not included. Percutaneous endoscopic gastrostomy was not feasible due to inadequate transillumination in 35 (32.7%) patients with advanced or recurrent ovarian cancer. 

Prior to admission to CT-PG, 27 (87%) patients had an unsuccessful or complicated attempt at percutaneous endoscopic gastroscopy (*n* = 25) and/or underwent surgery due to ileus (*n* = 3) with lysis of adhesions and stoma formation with or without bowel resection, which could not alleviate the obstruction symptoms. One patient, who previously underwent an endoscopic gastrostomy, was complicated with a transhepatic insertion of the G-tube.

### 3.1. Patient and Disease Characteristics 

The mean age at the time of bowel obstruction was 57 (range: 20–74) years. The mean BMI was 24.8 (range: 16–34.5) kg/m². The mean interval from the cancer diagnosis to bowel obstruction was 38.7 (range: 0–199) months.

All patients had peritoneal carcinomatosis and 21 (68%) had malignant ascites. For a mean of 7.3 days (range: 1–17) before the CT-PG insertion, paracentesis was performed with indwelling abdominal drain in 9 (29%) patients. Ascites volume was >500 mL in 8 patients; in the other 13 patients, free or loculated ascites was measured or estimated by CT-scan as ≤500 mL. An additional preprocedural paracentesis of pre-gastric loculated ascites was carried out for gaining safe access during CT-PG insertion in 1 patient.

Before gastrostomy, 17 of 31 (55%) patients had received ≥3 lines of chemotherapies; only 4 patients were chemotherapy-naive. Twenty-seven patients (87%) had recurrent disease, of which twenty-one (68%) were classified as platinum-resistant recurrence based on the progression-free interval of <6 months after the latest platinum-based chemotherapy. A total of 9 patients were undergoing chemotherapy and five were under maintenance therapy with a parp-inhibitor or bevacizumab. Table 1 provides an overview of the patients and disease characteristics. 

### 3.2. Success Rate and Symptom Relief 

**Figure 2:**
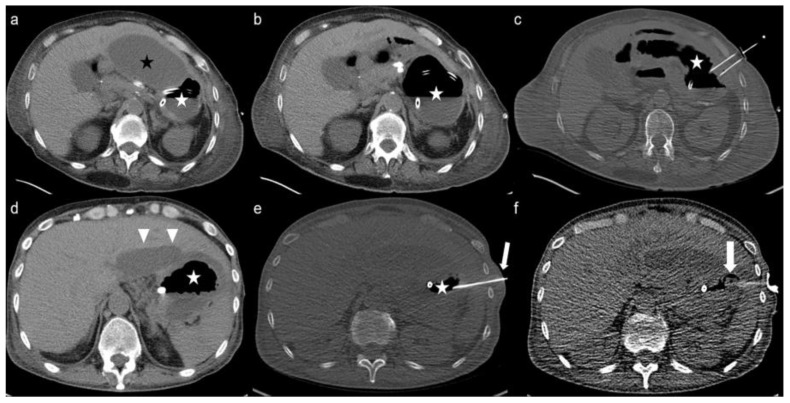
Axial CT and CT-fluoroscopy images illustrate the management of two complex cases in whom the endoscopic approach was not feasible: (**a**) perigastric accumulation of ascites *(black star)* constrains the punction field to the stomach *(white star)* and poses the risk of breakage; (**b**) after paracentesis, insufflation of the stomach using the nasogastric tube; (**c**) tacking the stomach to the anterior abdominal wall with gastropexy sutures; (**d**) image shows the kissing-spleen hepatomegaly *(triangles)* narrowing the punction field in another patient; (**e**) puncture of the stomach is only possible via 9th intercostal space *(arrow)*; (**f**) image demonstrating the successfully positioned G-tube with the inflated balloon in gastric lumen.

**Figure 3:**
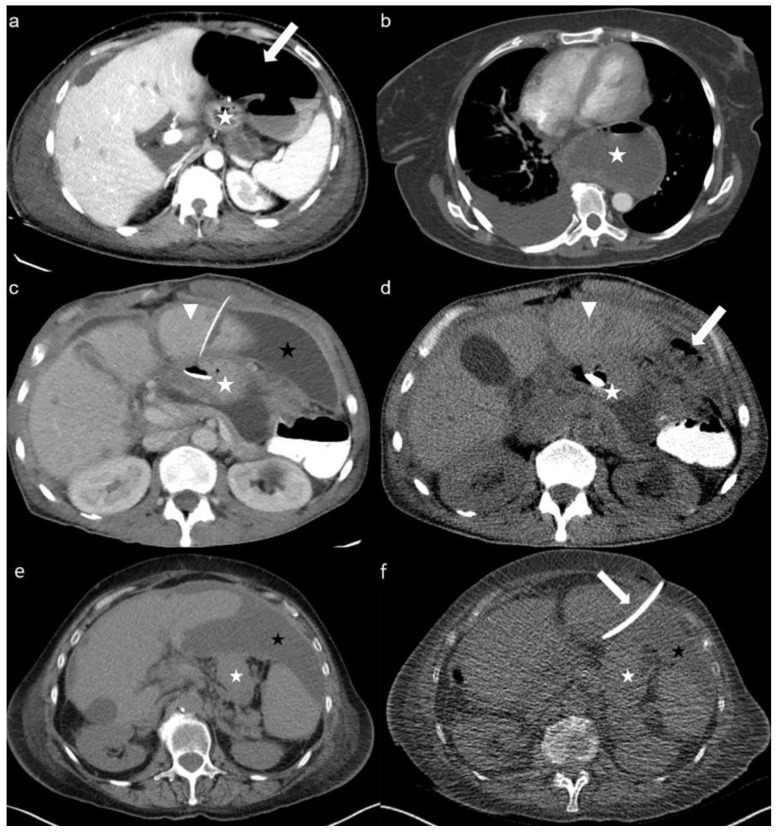
Sequential axial enhanced (**a**–**c**) and unenhanced (**d**) CT images demonstrate insufficient punction fields yielding unsuccessful CT-PG insertions in 4 patients: (**a**) Colonic interposition *(arrow)* ventral to the stomach *(star)*; (**b**) Hiatus hernia *(star)*; (**c**,**d**) the endoscopically inserted PEG tube is located in the gastric lumen *(white star)* and passes through the liver (*triangle*) as a complication of the previous endoscopic insertion. After paracentesis, insufficient punction field due to hepatomegaly *(triangle)* and colonic interposition *(arrow)* in the same patient; (**e**,**f**) presence of excessive haemorrhagic ascites *(black star)* despite multiple paracentesis and drainage *(arrow)*.

CT-guided gastrostomy could be successfully placed in 25 of 31 patients, yielding a success rate of 81% without any life-threatening interventional complications. CT-PG insertion was feasible in 19 of 25 (76%) patients with prior unsuccessful or complicated attempts of endoscopic insertion. Figure 2 demonstrates two complex cases managed with CT-PG insertion, in whom endoscopic insertion of the gastric tube was not feasible. Insufficient punction fields due to colonic or hepatic interpositions, hiatus hernia, and the presence of excessive haemorrhagic ascites ventral to the stomach despite several paracentesis led to 6 (19%) unsuccessful CT-PG insertions (Figure 3). 

The mean length of stay following a successful CT-PG insertion was 10 days (range: 1–50 days). A total of 20 out of 25 (80%) patients with successful CT-PG had considerable relief of nausea and vomiting. After the alleviation of symptoms, patients could intake small amounts of fluids and ice cubes, and 12 could also resume a low-residue diet. A total of 5 patients (20%) did not experience satisfactory symptom relief; 3 of them were associated with tube dysfunctions necessitating the upsizing of the catheter with 20Fr gastrostomy tubes. Only 1 patient underwent surgery due to persisting symptoms; however, achieving surgical correction was also not possible. 

As a moderate (grade 3) complication, we observed an intraperitoneal extragastric misplacement of the G-tube, which was recognised upon its nonfunctioning and replaced under CT-guidance. Within 1 month, we observed one accidental and one spontaneous dislodgement of the tube without signs of peritoneal contamination and replaced the tube immediately. A patient, in whom gastropexy sutures could not be applied due to a narrow punction field, developed clinical symptoms of mild peritonitis within the first 30 days after the procedure. The CT scan revealed that the stomach had no contact with the anterior abdominal wall, with localised perigastric fluid and air collection suggestive of leakage. After readjusting the tube endoscopically and the percutaneous drainage of the collection under antibiotic therapy, the patient recovered completely.

As a grade 1 complication, we observed local bleeding at the punction site in two patients, which could be managed with local compression without any further interventions or blood transfusion. Local skin irritations were documented in 4 (16%) patients, with one peristomal leakage requiring tube replacement. 

As late complications, five (20%) tube dislocations were documented, which were managed endoscopically, CT-guided or bed-side after 30 days following the initial CT-PG placement. 

With regard to electrolyte disturbances, 5 patients already had hypokalaemia (Kalium <3.5 mmol/L) before the insertion of CT-PG as a result of persistent vomiting. Additionally, 8 (32%) patients subsequently developed hypokalaemia (mean: 2.9 mmol/L range: 2.7–3.3 mmol/L) as a minor metabolic complication following CT-PG insertion. All patients were on total parenteral nutrition before and after CT-PG placement. 

### 3.3. Survival

Overall, 25 (81%) patients could be discharged with homecare facilities or placed in hospice/palliative care wards to continue end-stage palliation. In a median follow-up of 14 days (range 1–420 days), we observed 16 (52%) deaths in all of the intention-to-treat population, and 8 of 16 deaths occurred within 30 days following the procedure. The mean survival time was 44.4 days (range: 1–420 days) after the procedure. Including the patients who were lost to follow-up, we identified 1-month, 3-month and 6-month survival rates of 35% (11 of 31), 19% (6 of 31), and 3% (1 of 31), respectively. 

Chemotherapy could be administered in 7 of 25 (28%) patients following a successful CT-PG insertion with three platinum-based regimes and four platinum-free chemotherapy. One patient with a primary diagnosis of ovarian cancer with peritoneal carcinomatosis could receive six cycles of neoadjuvant chemotherapy with Carboplatin AUC2 and Paclitaxel 60 mg/m² q7d. In the interval surgery, macroscopical complete tumour resection was not achievable. The patient received further chemotherapy with regular changes and care of the tube. All other patients were managed according to best-supportive-care principles due to their high frailty and were placed in the hospice/palliative wards or discharged with homecare facilities shortly after the alleviation of symptoms. 

### 3.4. Literature Review

The review of selected studies revealed 289 patients with small bowel obstruction undergoing endoscopic, radiological, or surgical decompressive gastrostomies. Table 2 provides an overview of the selected studies. Overall, 147 (50.8%) of these patients had ovarian cancer. In other patients, MBO was associated with gastric, cervical, endometrial, breast, colorectal, pancreatic, or appendiceal cancers. Only 81 of 289 (28%) decompressive gastrostomies were performed under CT guidance. A success rate of 82–100% was reported for all gastrostomies. The main reasons for referring to CT-PG were inadequate transillumination, peritoneal carcinomatosis, hepatosplenomegaly, interposed bowel loops, previous gastric operation, or obesity. Considering only three studies explicitly pertained to CT-guided insertion as the primary method of gastrostomy, 29 of 74 (39.1%) decompressive gastrostomies were carried out due to MBO associated with ovarian cancer [13,16,18]. Overall reported minor complications were mainly related to tube dysfunctions, dislodgement, and peristomal leakage requiring tube replacement and superficial skin infections with a rate of approximately 14–32%. One case of local peritonitis occurred due to the disconnection from the abdominal wall, which recovered after adjusting the tube under antibiotic therapy; a misplacement into the colon and a deep skin infection requiring surgical treatment were noted under CT-fluoroscopy guidance by Spelsberg et al. [18]. Symptom relief was not evaluated within the studies pertaining to CT-guided gastrostomies but was generally estimated as 77.4–92% for decompressive gastrostomies with an improvement in the quality of life in 64% of patients. Interpreting the survival results was impossible due to the diverse populations of the studies. 

## 4. Discussion

This descriptive study highlights the feasibility of CT-PG in peritoneal cancer as a minimally invasive technique, particularly when endoscopic gastrostomy is unfeasible due to the loss of transillumination related to anatomical disruptions and tumour deposits encasing the stomach. CT-PG insertion was successful in 81% of all patients and 76% of patients with previous unsuccessful or complicated attempts at endoscopic gastrostomy.

Our experience shows effective symptom control in 80% of patients without any severe interventional complications. Since parenteral hydration does not palliate symptoms like thirst and mouth dryness, patients could still enjoy liquids after the resolution of emesis and some patients could even tolerate a low-fibre diet and decompress digested content over the valve when necessary.

CT-PG was not feasible due to colonic or hepatic interpositions, hiatus hernia, and the presence of excessive haemorrhagic ascites in 19% of patients. However, ascites drainage or the preprocedural paracentesis of ascites facilitated safe access in some cases. Indeed, it is recommended to evacuate the large volume ascites before the procedure and repeat paracentesis if ascites reaccumulates to ensure the safe maturation of the gastrostomy tract [21].

Gynaecological cancer patients with MBO are usually heavily pre-treated, frail patients with a life expectancy of approximately four months [22]. Decompressive gastrostomy eliminates the requirement for a nasogastric tube, facilitates the resumption of fluids, and makes caregiving at home or hospice less distressing for a terminal patient [23]. Endoscopic gastrostomy provides adequate symptom control in 84–100% of patients with a success rate of 86–100% [24,25,26]. Nevertheless, the endoscopic approach was unfeasible in 32.7% of patients due to anatomical constraints and lack of transillumination in our series. Zucchi et al. reported limitations in endoscopic gastrostomy in approximately 10% of patients with abdominopelvic carcinomatosis related to gastrointestinal or gynaecological cancers [17].

CT-fluoroscopy provides a rapid recognition of anatomy in previously operated advanced cancers with an eventual reduction in complication rates [27]. Minimizing the risk of complications is particularly important in these frail patients within the scope of palliative interventions. Considering that these patients were otherwise not treatable and the symptom relief after CT-PG was mostly adequate, this technique should be used more frequently within the palliative management of MBO. On the other hand, an initial endoscopic evaluation of the upper gastrointestinal tract is advisable to identify other gastric pathologies, such as peptic ulcer or gastric-outlet obstruction, which can eventually change the management of the case [28].

The literature offers various studies on decompressive endoscopic or fluoroscopic gastrostomy in MBO. However, the use of CT-guided gastrostomy with decompressive purposes in ovarian cancer has very rarely been reported. Our literature review revealed that the CT-guided technique is mainly utilised in a limited number of patients as a secondary method if the endoscopic approach fails. Albrecht et al. reported 45 patients undergoing decompressive CT-PG following an unsuccessful endoscopic gastrostomy; 20 patients had peritoneal carcinomatosis; and 14 patients were associated with ovarian cancer. They showed successful insertion in 82% of decompressive gastrostomies compared to a success rate of 91% in gastrostomies for nutritional purposes [16]. Spelsberg et al. described decompressive CT-PG in 13 patients with peritoneal carcinomatosis, including 7 patients suffering from ovarian cancer [18]. In the prospective analysis by Zucchi et al., 158 consecutive patients with MBO related to gastrointestinal and gynaecological cancers were mainly managed with an endoscopic technique. They indicated the necessity of sonographic guidance in 8 (5%) and CT-guided insertion in 4 (2.5%) patients due to improper transillumination or compression of the stomach [17].

The rate of severe complications including death, haemorrhage, peritonitis, and aspiration ranges from 0.4% to 2.5% [29,30]. We documented an intraperitoneal extragastric misplacement of the tube, two early dislodgements, and one leakage associated with local peritonitis. Gastrostomy placement in the presence of excessive ascites is usually demanding and carries the risk of peritonitis; hence, serial controls are recommended to evaluate and decompress the accumulated ascites to ensure the maturation of the stoma track [31]. Moreover, the use of gastropexy sutures can also reduce the risks of breakage and intestinal leakage in cases with marked ascites, which could not be applied due to the narrow punction field in our case [32]. Other early and late complications were mainly associated with tube dysfunctions due to occlusion or dislodgement requiring CT-guided or endoscopic reinsertion.

Our study population represents a heavily pre-treated group of peritoneal cancer patients, with 55% having received ≥3 lines of chemotherapy. Following a successful CT-gastrostomy, 28% patients could receive further chemotherapy, while others were placed in hospice or discharged with homecare facilities to continue their end-stage palliation. In the analysis of 94 patients with ovarian cancer submitted to endoscopic gastrostomy due to MBO by Pothuri et al., 29 (31%) patients could receive chemotherapy after PEG placement. Nevertheless, the complete resolution of obstruction and removal of PEG was possible in only 4 patients [24]. The reported median-OS was 8 weeks, and 85% of patients died at home or in hospice care. In line with these results, the mean survival including patients who were lost to follow-up after discharge was 44.4 days in our study, and 81% of patients could be discharged to continue end-stage palliation. In the study by Zucchi et al., 9.8% of patients with abdominal-pelvic carcinomatosis due to advanced gynaecological or gastrointestinal cancers could undergo salvage chemotherapy following symptom palliation with decompressive gastrostomy [17]. These findings underline that the objective of decompressive gastrostomy is to provide palliative end-of-life care in the majority of patients; however, the application of salvage chemotherapy or first-line therapy in the primary setting may be possible in selected patients. Nevertheless, the best predictor of treatment effectiveness in MBO is the sensitivity of the tumour to platinum-based chemotherapy [33].

The primary limitation of our study is its retrospective origin, which naturally hampers the assessment of patients’ experiences concerning partial or complete alleviation of symptoms. Symptom resolution was documented on the basis of self-reported physical symptoms during multidisciplinary clinical assessments by attending physicians and the palliative team. Patients were not systematically interviewed for QoL assessment using a validated distress scale, which could reveal the variation in intensity and frequency of symptoms after the procedure. Moreover, due to the terminal status of disease, and thus the limited follow-up in the majority of patients, complications might also be under-recognised. Nevertheless, despite the limited numbers, our study represents one of the largest series specifying the CT-guided approach, especially in otherwise unfeasible gastrostomies in MBO associated with ovarian cancer.

## 5. Conclusions

In summary, based on our results and our interpretation of the literature, we can conclude that CT-guided percutaneous gastrostomy is a well-tolerated procedure for the palliation of ovarian cancer-associated small bowel obstruction. We believe that this minimally invasive technique should be highlighted as a routine instrument within the palliative management of MBO, particularly in complex patients with altered anatomy related to peritoneal carcinomatosis and previous surgeries. A clinical registry should be implemented for MBO in gynaecological cancers, which may serve as a unique tool to increase our understanding concerning the effect of various interventions on clinical outcomes and the patients’ quality of life.

## Figures and Tables

**Table 1:** Characteristics of the study population undergoing CT-PG due to MBO associated with ovarian cancer.

Histology	*n* = 31
HGSOC	22 (71%)
LGSOC	5 (16%)
Mucinous	3 (10%)
Clear cell	1 (3%)
Status of disease	
Primary diagnosis	4 (13%)
Recurrent disease	27 (87%)
Platinum-resistant	21 (68%)
Platinum-sensitive	5 (16%)
Not assessable	1 (3%)
Malignant ascites	
none	10 (32%)
≤500 mL	13 (42%)
>500 mL	8 (26%)
Prior lines of Chemotherapies	
none	4 (13%)
1–2	10 (32%)
≥3	17 (55%)
Interventions for MBO prior to CT-PG insertion	
Attempt for PEG	25 (81%)
Surgery for ileus	3 (10%)
None	4 (13%)
Chemotherapy after successful CT-PG	n = 25
Platinum-based	3 (12%)
Platinum-free	4 (16%)
None	18 (72%)
Procedure-related complications within 1 month	
Grade 4–5	0
Grade 3	4 (16%)
Misplacement	1 (4%)
Dislodgement	2 (8%)
Leakage	1 (4%)
Grade 1–2	6 (24%)
Local bleeding	2 (8%)
Peristomal leakage	1 (4%)
Local skin irritations	4 (16%)

HGSOC: High-grade serous ovarian cancer; LGSOC: Low-grade serous ovarian cancer; PEG: percutaneous endoscopic gastrostomy.

**Table 2:** Review of the literature.

Author, Year	Albrecht, 2017 **[16]**	Zucchi, 2016 **[17]**	DeEulis, 2015 **[19]**	Spelsberg, 2013 **[18]**	Brooksbank, 2002 **[20]**	Sanchez, 1992 **[13]**
Patients, N	102	158	6	101	51	22 **
Indications (N of patients)	feeding (57); decompression (45)	decompression (158)	decompression (6)	feeding (87); decompression (14)	decompression (51)	feeding (7); decompression (15)
Ovarian cancer N (%)	14 (31.1%)	96 (60.7%)	6 (100%)	7 (6.9%)	16 (31.3%)	8 (36.3%)
The primary route of access in the study (N)	CT-guided with simultaneous endoscopy (98)	Endoscopic (PEG: 142 or PEJ: 14)	Radiologic (2) Endoscopic (2) Surgical (1)	CT-fluoroscopy with or without simultaneous endoscopy (101)	Endoscopic (46) Surgical (4)	CT-guided (22)
CT-guided decompressive gastrostomy, N	45	CT-PEG: 3 CT-PEJ: 1	2	14	1	15
The main reasons for referring to CT-PG (N)	inadequate transillumination (73), peritoneal carcinosis (20), obstructed passage (9). Upper GIS endoscopy was generally attempted in all patients	stomach dislocation (2), gastric tubularization (1)	n.a.	severe pharyngeal or oesophagal obstruction (30), recent pharyngeal surgery (20), peritoneal carcinosis (13), inadequate transillumination (6)	not clearly defined: Inability to distend the stomach and transilluminate the abdominal wall (1), oesophageal obstruction (1)	peritoneal tumour mass (6), large or low-lying liver (4), small gastric remnant (2), interposed bowel (n = 2), and prominent overlying ascites (n = 2).
Patients with ascites, N	n.a.	n.a.	3	n.a.	n.a	2
Paracentesis, N	n.a.	n.a.	3	n.a.	n.a	n.a
Success rate (all)	87.3% * f-PEG: 91.2% d-PEG: 82.2%	89.8 %	5 out of 6 patients	88% §	96%	100%
Symptom relief (N)	n.a.	77.4 % experienced relief within 2 days; 64 % (16 of 25) exhibited improvement of QoL	symptoms improved (6); tolerated clear liquids, pureed and soft foods (5); gastric tube removal (1)	n.a.	92% experienced symptom relief; resume oral soft food and fluid intake.	n.a.
Complications (N)	Dislocation (2); Minor complications (tube dysfunction, local bleeding, minimal leakage, local skin infection) in 13 of 102 patients	Peristomal infection (14%), obstruction (8.4%), gastric leakage from ostomy (1.4%), gastric bleeding (2.1%), PEG displacement (2.1%), failure (1)	No patients in this series experienced major or minor complications related to gastrostomy placement or function.	No mortality; misplacement into the colon (1), local peritonitis (1), deep skin infection (1), dislodgement (17), peristomal leakage (7), superficial skin infection (6), tube obstruction (2)	Pain at insertion site, haematoma in the abdominal wall (1), Excoriation of the skin (2), leakage of gastric contents (4), Tube blocking/dislodgement (4).	No major complications; Replacement due to kink (2) at 45 and 53 days; catheter fracture (1) at 14 days.
Chemotherapy after gastrostomy	n.a.	9.8 % underwent salvage chemotherapy	1 patient: 5th line cisplatin followed by 6th line topotecan	n.a.	-	n.a.

PEG: percutaneous endoscopic gastrostomy; PEJ: percutaneous endoscopic jejunostomy; * Reasons the procedures failed or were aborted included the following: stomach or proximal jejunum covered by a dilated colon or left lobe of the liver (*n* = 11); intramural gastric abscess–preoperative diagnosis with CT (*n* = 1); and vomiting and aspiration during the intervention (*n* = 1); ** Previous attempts for endoscopic G-Tube placement in 13 patients; fluoroscopic insertion failed in 17 patients.; ^§^ The reasons for not achieving success were the following: the stomach or proximal jejunum was covered by dilated colon or left lobe of the liver (*n* = 8), or the residual stomach was too small after a partial gastrectomy to permit the puncture (*n* = 4).

## Data Availability

The data that support the findings of this study are available from the corresponding author (EC), upon reasonable request.
